# Community-associated *Clostridium difficile* Infections, Monroe County, New York, USA

**DOI:** 10.3201/eid1803.102023

**Published:** 2012-03

**Authors:** Ghinwa Dumyati, Vanessa Stevens, George E. Hannett, Angela D. Thompson, Cherie Long, Duncan MacCannell, Brandi Limbago

**Affiliations:** University of Rochester, Rochester, New York, USA (G. Dumyati, V. Stevens);; State University of New York at Buffalo, Buffalo, New York, USA (V. Stevens);; New York State Department of Health, Albany, New York, USA (G.E. Hannett);; Centers for Disease Control and Prevention, Atlanta, Georgia, USA (A.D. Thompson, C. Long, D. MacCannell, B. Limbago)

**Keywords:** Clostridium difficile, bacteria, community-associated infections, health care–associated infections, strain typing, antimicrobial drug resistance

## Abstract

Judicious use of antimicrobial drugs will reduce infections.

## Medscape CME Activity

Medscape, LLC is pleased to provide online continuing medical education (CME) for this journal article, allowing clinicians the opportunity to earn CME credit.

This activity has been planned and implemented in accordance with the Essential Areas and policies of the Accreditation Council for Continuing Medical Education through the joint sponsorship of Medscape, LLC and Emerging Infectious Diseases. Medscape, LLC is accredited by the ACCME to provide continuing medical education for physicians.

Medscape, LLC designates this Journal-based CME activity for a maximum of 1 *AMA PRA Category 1 Credit(s)*^TM^. Physicians should claim only the credit commensurate with the extent of their participation in the activity.

All other clinicians completing this activity will be issued a certificate of participation. To participate in this journal CME activity: (1) review the learning objectives and author disclosures; (2) study the education content; (3) take the post-test with a 70% minimum passing score and complete the evaluation at www.medscape.org/journal/eid; (4) view/print certificate.

**Release date: February 22, 2012; Expiration date: February 22, 2013**

## Learning Objectives

Upon completion of this activity, participants will be able to:

- Describe the relative burden of and potential risk factors for community-associated (CA) disease, based on a 6-month surveillance program for laboratory-diagnosed *Clostridium difficile* infection (CDI) cases in Monroe County, New York, in 2008
- Compare clinical characteristics of patients with CA disease with those having acquired CDI in other settings, based on the surveillance program
- Describe clinical implications of this study, including prevention strategies for CA-CDI and comparison of *C. difficile* recovery rates between refrigerated stool swabs and frozen stools

## CME Editor

**Thomas J. Gryczan, MS,** Technical Writer/Editor, *Emerging Infectious Diseases. Disclosure: Thomas J. Gryczan, MS, has disclosed no relevant financial relationships.*

## CME Author

**Laurie Barclay, MD,** freelance writer and reviewer, Medscape, LLC. *Disclosure: Laurie Barclay, MD, has disclosed no relevant financial relationships.*

## Authors

*Disclosures:* ***Ghinwa Dumyati, MD; Vanessa Stevens, PhD; George E Hannett, MS; Angela D. Thompson, MS; Cherie Long, MPH; Duncan MacCannell, PhD;**** and ****Brandi Limbago, PhD,*** *have disclosed no relevant financial relationships.*
