# Supplementary material for: Community-associated Clostridium difficile Infections, Monroe County, New York, USA
Source: Emerg Infect Dis. 2012 Mar;18(3):392–400. doi: 10.3201/eid1803.102023 (PMC3309637; doi:10.3201/eid1803.102023)
Supplement: Related texts

# Research article: Community-associated *Clostridium difficile* Infections, Monroe County, New York, USA

**DOI:** 10.3201/eid1803.102023

**Authors:** Ghinwa Dumyati, Vanessa Stevens, George E. Hannett, Angela D. Thompson, Cherie Long, Duncan MacCannell, Brandi Limbago

**Affiliations:** University of Rochester, Rochester, New York, USA (G. Dumyati, V. Stevens);; State University of New York at Buffalo, Buffalo, New York, USA (V. Stevens);; New York State Department of Health, Albany, New York, USA (G.E. Hannett);; Centers for Disease Control and Prevention, Atlanta, Georgia, USA (A.D. Thompson, C. Long, D. MacCannell, B. Limbago)

## Abstract

We conducted active sentinel surveillance in Monroe County, New York, USA, to compare incidence of community-associated *Clostridium difficile* infections (CA-CDIs) with that of health care–associated infections (HA-CDIs) and identify exposure and strain type differences between CA and HA cases. Patients positive for *C. difficile* toxin and with no documented health care exposure in the previous 12 weeks were defined as possible CA case-patients. Patients with onset in a health care setting or recent health care exposure were defined as HA case-patients. Eighteen percent of CDIs were CA; 76% were in persons who reported antimicrobial drug use in the 12 weeks before CDI diagnosis. Strain type distribution was similar between CA and HA cases; North American pulsed-field 1 was the primary strain (31% CA, 42% HA; p = 0.34). CA-CDI is an emergent disease affecting patients recently exposed to antimicrobial drugs. Community strains are similar to those found in health care settings.

*Clostridium difficile* is an anaerobic, spore-forming, gram-positive bacillus that produces 2 major toxins (TcdA and TcdB). Illness caused by toxigenic *C. difficile* varies from mild diarrhea to fulminant disease and death. Infection occurs commonly in the health care setting because of concomitant exposure to the organism and antimicrobial drugs in patients with severe illnesses and concurrent conditions. Over the past several years, the incidence (*1*), severity and mortality rate (especially in elderly persons) (*2*), and treatment failure rate of *C. difficile* infection (CDI) (*3*) have increased. In addition, CDI has been more commonly observed in healthy persons often without known CDI risk factors (*4*).

The changing pattern of disease is in part being caused by the emergence of a new epidemic hypervirulent *C. difficile* strain identified as North American pulsed-field 1 (NAP1) by pulsed-field gel electrophoresis (PFGE), BI by restriction endonuclease analysis, and 027 by PCR ribotyping (*5*). NAP1 strains often demonstrate resistance to quinolones, and increased use of these drugs may provide a positive selection pressure for NAP1 relative to other strains (*6*). The incidence, risk factors, and mode of transmission of CDI in hospital-associated (HA) disease are well described. However, few studies have examined the role of the hypervirulent NAP1 strain, antimicrobial drugs, proton pump inhibitors (PPI), and foodborne transmission on the emergence of CDI (*7**–**19*).

To define the magnitude of CDI across the continuum of care (hospital, long-term care, and the community) and assess the relative incidence and possible risk factors for community-associated disease, a 6-month surveillance program for laboratory-diagnosed CDI cases was initiated in Monroe County, New York, USA, in 2008. This program was undertaken in 2 sentinel laboratories in preparation for population-based surveillance of CDI in several US states through the Emerging Infections Program of the Centers for Disease Control and Prevention (CDC). A secondary goal of this study was to compare *C. difficile* recovery rates between refrigerated fecal swab and frozen fecal specimens.

## Methods

### Setting

During March 1–August 31, 2008, surveillance officers reviewed all medical records associated with *C. difficile* toxin–positive fecal samples from 2 of 3 hospital laboratories in Monroe County, New York. These laboratories service long-term care facilities, doctors’ offices, and the inpatient population. Electronic inpatient and outpatient medical records for all patients with fecal samples positive for *C. difficile* toxin by enzyme immunoassay were reviewed.

### Case Definitions

Cases were subcategorized according to published surveillance definition guidelines (*20*). The date of CDI onset was defined as the date of the positive fecal test result, not the date of diarrhea onset. The date of diarrhea onset was not readily available in the electronic medical records. An incident case was defined as disease in a patient with a *C. difficile* toxin-positive fecal sample and no positive assay result in the preceding 8 weeks. Cases were considered recurrent if there was a positive assay result within 2–8 weeks of the most recent toxin-positive specimen. Positive assay results obtained within 2 weeks of the prior positive assay result were considered duplicates and excluded. Possible case-patients were also excluded if they had no documented diarrhea, an initial fecal sample representing recurrence of an episode before the start date of surveillance, were <12 months of age at the time of testing, or were not residents of Monroe County.

Cases were classified into 3 categories. The first category was health care facility onset (HCFO) cases. These cases were in CDI case-patients who had *C. difficile* toxin–positive fecal specimens obtained >48 hours of hospital admission or during residence in a long-term care facility.

The second category was community-onset health care–associated (CO-HCA) cases. These cases were in CDI case-patients who had *C. difficile* toxin–positive fecal specimens obtained <48 hours of hospital admission or as an outpatient and who had documented exposure to health care in the previous 12 weeks. Health care exposure was defined as a >24-hour stay in a hospital or 48 hours in an emergency department, residence in a long-term care facility, receipt of chronic hemodialysis, or regular hospital visits for intravenous infusion. No differentiation was made between case-patients with disease onset within 4 weeks of discharge and those with disease onset 4–12 weeks after discharge from the health care facility (i.e., indeterminate case) (*20*). Cases in the HCFO and CO-HCA categories were referred to as health care–associated (HA) CDI. 

The third category was potential community-associated (potential CA) cases. These cases were in CDI case-patients who had *C. difficile* toxin–positive fecal specimens obtained within 48 hours of hospital admission or in an outpatient setting and who had no documented health care exposure in the 12 weeks before the positive test result. Potential CA case-patients were interviewed to confirm the lack of health care exposures, and to assess contact with health care personnel, medication use, travel, food consumption, and exposure to animals in the previous 12 weeks. Potential CA case-patients who were interviewed and reported exposure to health care in the previous 12 weeks during the interview were reclassified as having CO-HCA; those who denied such exposures were confirmed as having CA-CDI. Patients who died, could not be reached, did not speak English, refused consent, or whose doctor refused consent were classified as having probable CA-CDI.

Information was obtained on demographics; previous positive assay results; dates of admission to acute-care facilities, emergency departments, and long-term care facilities; inpatient mortality rates; and CDI complications, such as toxic megacolon, renal failure, or intensive care unit admission. The study was approved by the institutional review boards at CDC, the University of Rochester Medical Center, Rochester General Hospital, and the New York State Department of Health.

### Laboratory Methods

Fecal samples (unformed) were tested at clinical laboratories for *C. difficile* toxin by using the Premier Toxins A & B enzyme immunoassay (Meridian Bioscience, Inc., Cincinnati, OH, USA). Aliquots of fecal specimens were stored at −20°C until cases were reviewed. All available fecal samples of patients classified as potential CA and a random sample of the CO-HCA and HCFO (1 of each classification each week) were submitted to the New York State Public Health Laboratory (Wadsworth Center Laboratory, Albany, NY, USA) for culture. For 31 randomly chosen stool specimens, an additional specimen was obtained by using a culture swab (Copan 159C or similar). To avoid germination of *C. difficile*, no enrichment or anaerobic transport media were used. Fecal swab specimens were stored at 4°C to compare the recovery rate from a swab stored at 4°C with that for a frozen fecal aliquot. Both specimen types were stored for several weeks before shipment and were shipped on dry ice.

### Culture and Molecular Characterization Methods

Fecal specimens were placed on cycloserine-cefoxitin fructose agar plates and incubated at 35°C under anaerobic conditions for 96 hours. Culture-negative fecal samples and swab specimens were treated by using alcohol shock (*21*) and recultured on cycloserine-cefoxitin fructose agar containing 0.1% sodium taurocholate (*22*). Plates were examined daily for characteristic colonies. Isolates were shipped to CDC for molecular characterization, which included PCR for binary toxin and major *tcdC* gene deletions, toxinotyping (*22**,**23*), and *Sma*I PFGE. PFGE banding patterns were analyzed by using BioNumerics version 5.10 (Applied Maths, Austin, TX, USA) and compared with the CDC *C. difficile* database (*24*). NAP types were assigned to patterns with ≥80% similarity to established NAP clusters. MICs for clindamycin, metronidazole, moxifloxacin, levofloxacin, and vancomycin were determined by using the agar dilution method, and results were interpreted by using the Clinical and Laboratory Standard Institute M11-A7 breakpoint criteria (*25*). For surveillance purposes only, levofloxacin MICs were interpreted by using criteria for moxifloxacin, and vancomycin MICs were interpreted by using criteria for *Staphylococcus aureus*.

### Statistical Analysis

All statistical analyses were performed with SAS version 9.1 (SAS Institute, Cary, NC, USA) assuming a 2-tailed α of 0.05. Univariate analysis was conducted to summarize the demographic and clinical characteristics of case-patients. Bivariate analyses were used to compare these characteristics across preliminary and final classifications by using Wilcoxon rank sum, χ^2^, and Fisher exact tests as appropriate.

## Results

### Surveillance

During the study, 366 incident CDI cases were identified after excluding 558 *C. difficile* toxin–positive stool assays for patients who did not meet eligibility criteria. The distribution of cases is shown in Figure 1. Of these cases, 196 (54%) were categorized as HCFO and 170 (46%) as CO cases. Eighty-three cases (22% of all cases) were potentially community-associated, 58 (72%) of patients with these cases were interviewed, and 16 (20%) cases were reclassified as CO-HCA. Overall, 67 cases (18% of all cases) were classified as CA; 42 of these cases were confirmed by interview and 25 were considered probable CA. Review of available electronic inpatient and outpatient records for the probable case-patients showed no previous exposure to health care. Therefore, we believe that most cases were truly CA. The probable and definite CA case-patients had similar ages, race distributions, and outcomes.

**Figure 1:** Classification of cases of *Clostridium difficile* infections, Monroe County, New York, USA, March 1–August 31, 2008. CO-HCA, community onset–health care associated; HO, hospital onset; LTCFO, long-term care facility onset.

### Clinical Characteristics

Clinical characteristics of CDI cases by epidemiologic classification are shown in Table 1. Compared with HCFO and CO-HCA case-patients, CA case-patients (confirmed and probable) were younger (median age 53 vs. 78 and 69 years, respectively; p<0.001). Illness among CA case-patients was milder; only 13 (19%) of patients required hospitalization compared with 39 (38%) CO-HCA case-patients (p = 0.02) (Table 2). Duration of hospitalization was 7.0 days vs. 3.5 days (p = 0.06) for CO-HCA and CA case-patients, respectively. None of the hospitalized CA case-patients died or had any complications. Laboratory confirmation of recurrence was documented in 22% of the HCFO and CO-HCA case-patients and 12% of the CA case-patients (Table 2).

**Table 1:** Characteristics of case-patients with CDI, Monroe County, New York, USA, March 1–August 31, 2008*

Characteristic	HCFO	CO-HCA	CA†	p value
Total	196	103	67	ND
Demographic				ND
Female sex	116 (59)	65 (63)	41 (61)	0.75
Median age, y (SD)	78 (17)	69 (17.4)	53 (20.8)	<0.001
Race				0.75
White	142 (83)	87 (87)	45 (82)	ND
Black	25 (14)	12 (12)	8 (14)	ND
Other‡	5 (3)	1 (1)	2 (4)	ND

*Values are no. (%) except as indicated. CDI, *Clostridium difficile* infection; HCFO, health care facility onset; CO-HCA, community onset–health care associated; CA, community associated; ND, not determined. †Definite and probable CA cases. ‡Asian, American Indian/Alaska Native, and Native Hawaiian/Pacific Islander.

**Table 2:** Outcomes for case-patients with CDI, Monroe County, New York, USA, March 1–August 31, 2008*

Characteristic	HCFO	CO-HCA	CA†	p value
Complications				
Hospitalized for CDI	NC	39 (38)	13 (19)	0.02
Median length of stay, d (SD)	NC	7 (17)	4 (11)	0.06
Outcome‡				
Survived	158 (80)	89 (86)	44 (66)	ND
Died	23 (12)	9 (9)	0 (0)	ND
Unknown	15 (8)	5 (5)	23 (34)	ND
Deaths caused by CDI				
Yes	7/23 (30)	3/9 (33)	NC	ND
No	3/23(13)	3/9 (33)	NC	ND
Unknown	13/23 (57)	3/9 (33)	NC	ND
Laboratory-confirmed recurrence (>1)	43 (22)	23 (22)	8 (12)	0.17

*Values are no. (%) positive or no. positive/no. tested (%) except as indicated. CDI, *Clostridium difficile* infection; HCFO, health care facility onset; CO-HCA, community onset–health care associated; CA, community associated; NC, not compared, ND, not determined. †Definite and probable CA cases. ‡For hospitalized patients, death occurred in the hospital. For nonhospitalized patients, death occurred 8 weeks after a positive *C. difficile* assay result.

### Interview Results

Forty-two case-patients were confirmed as CA-CDI case-patients by interview. Median (SD) age of CA-CDI case-patients was 53 (22.6) years. CA case-patients appeared healthy, and only 30% of case-patients had an underlying illness that required regular physician visits. Severity of reported symptoms varied; the median number of bowel movements per day was 10 (range 0–50 movements), and 1 patient had syncope before diarrhea onset. Other than diarrhea, 86% of patients reported abdominal pain, 48% nausea, 40% fever and 17% bloody feces. Average time from diarrhea onset to diagnosis was 14 days (range 0–92 days). Clinical recurrence requiring a repeat course of treatment was reported by 21%; some recurrences were treated without laboratory confirmation.

Medication and health care exposures of CA case-patients in the 12 weeks before CDI are shown in Table 3. Thirty-two (76%) of the interviewed case-patients reported using antimicrobial drugs, and 20% took >1 type of drug. The most commonly taken drugs were penicillins, followed by clindamycin and cepholosporins (Table 3). Indications for antimicrobial drug use included upper respiratory or ear infection (26%), bronchitis/pneumonia (17%), and dental abscess treatment or dental prophylaxis (17%). Twenty-six percent of CA case-patients reported using PPIs, and only 2% reported using H_2_ blockers.

**Table 3:** Possible exposures to medications and health care during 12 weeks before diagnosis of CA-CDI in 42 patients, Monroe County, New York, USA, March 1–August 31, 2008*

Exposure	No. (%)
Medication†	
Antimicrobial drugs	32 (76)
Penicillins	12 (31)
Clindamycin	7 (18)
Cephalosporins	5 (13)
Quinolones	5 (13)
Macrolides	4 (10)
Sulfa	3 (8)
Metronidazole	2 (5)
H_2_ blockers	1 (2)
PPI	11 (26)
Health care†	
None	5 (12)
Outpatient visit	35 (83)
Physician office	29 (69)
Dentist	13 (31)
Emergency department visit	6 (14)
Visited a hospital or LTCF	9 (21)
Health care–related job	2 (5)

*CA-CDI, community-associated *Clostridium difficile* infection; PPI, proton pump inhibitor; LTCF, long-term care facility. †Multiple exposures could be reported in the 12 weeks before CDI.

Of the 42 interviewed CA case-patients, 35 (83%) reported receiving care at an outpatient office in the 12 weeks before the *C. difficile* toxin–positive test result; 13 (31%) received care only at a dental office. Nine (21%) case-patients reported visiting health care facilities without receiving care in the 12 weeks before diagnosis. Five (12%) case-patients reported no exposure to any outpatient or inpatient health care facility. Eight (19%) case-patients did not report exposure to a health care facility or to antimicrobial drugs.

### Isolates

Of 145 fecal specimens cultured, 127 (87%) grew *C. difficile*. The recovery rates for HA and CA samples were 93% and 85%, respectively. The recovery of *C. difficile* was the same (80%) from fecal swab specimens and aliquots (100% agreement). One hundred nineteen *C. difficile* isolates underwent molecular and antimicrobial drug susceptibility testing. Six toxinotypes were identified; ≈50% of the isolates in each of the 3 epidemiologic classifications were wild-type toxinotype 0 (Table 4). Of the other variants, toxinotype III was the most commonly identified in all 3 epidemiologic classifications. Toxinotype V, a strain reported in food animals (*7**,**26*) was identified in 2.5%–5% of the samples and varied depending on the epidemiologic classification.

**Table 4:** Toxinotypes of strains from case-patients with CDI, Monroe County, New York, USA, March 1–August 31, 2008*

Characteristic	HCFO, no. (%)	CO-HCA, no. (%)	CA, no. (%)	Total, no. (%)
Total	41	40	38	119
Toxinotype				
0	20 (48.8)	22 (55.0)	17 (44.7)	59 (49.6)
III	19 (46.3)	12 (30.0)	18 (47.4)	49 (41.2)
V	1 (2.4)	1 (2.5)	2 (5.3)	4 (3.4)
IX/XXIII	0	3 (7.5)	0	3 (2.5)
XII	1 (2.4)	1 (2.5)	0	2 (1.7)
XIV/XV	0	0	1 (2.6)	1 (0.8)
Nontoxigenic	0	1 (2.5)	1 (0.0)	1 (0.8)

*CDI, *Clostridium difficile* infection; HCFO, health care facility onset; CO-HCA, community onset–health care associated; CA, community associated.

The PFGE types of 68% of the isolates were previously named NAP types; the remaining types were classified into 20 different and unnamed PFGE types (Table 5). The primary PFGE strain type among the 3 epidemiologic classes was NAP1, characteristically toxinotype III and carrying binary toxin and an 18-bp deletion in the *tcdC* gene. Three isolates identified as NAP1 by PFGE differed from the epidemic strain by having toxinotype IX/XIII and lacking the 18-bp deletion. Two of the strains from the CA isolates had characteristics that were typical of NAP1 but were <80% related to NAP1 by PFGE; these strains were classified as NAP1-related. NAP8, a strain associated with toxinotype V, was seen exclusively in CA isolates and at low numbers. NAP7 and NAP8 toxinotype V strains have been isolated from food animals in the United States (*7**,**26*).

**Table 5:** PFGE typing of *Clostridium difficile* from case-patients with CDI, Monroe County, New York, USA, March 1–August 31, 2008*

PFGE type	HCFO, no. (%)	CO-HCA, no. (%)	CA, no. (%)	Total, no. (%)
NAP1	19 (46.4)	14 (35.0)	12 (31.6)	45 (37.8)
NAP1-related	0	0	2 (5.3)	2 (1.7)
NAP2	2 (4.9)	4 (10.0)	1 (2.6)	7 (5.9)
NAP4	1 (2.4)	0	1 (2.6)	2 (1.7)
NAP5	2 (4.9)	6 (15.0)	0 (0.0)	8 (6.7)
NAP6	3 (7.3)	1 (2.5)	3 (7.9)	7 (5.9)
NAP7	1 (2.4)	1 (2.5)	1 (2.6)	3 (2.5)
NAP8	0	0	1 (2.6)	1 (0.8)
NAP10	0	1 (2.5)	1 (2.6)	2 (1.7)
NAP11	2 (4.9)	0	1 (5.3)	4 (3.4)
Unnamed	11 (26.8)	13 (32.5)	14 (36.8)	38 (31.9)
Total	41	40	38	119

*PFGE, pulsed-field gel electrophoresis; CDI, *Clostridium difficile* infection; HCFO, health care facility onset; CO-HCA, community onset–health care associated; CA, community associated; NAP, North American pulsed-field.

A comparison of antimicrobial drug susceptibilities of the epidemic strain NAP1/toxinotype III and other strains is shown in Table 6. The NAP1 epidemic strain is more resistant to quinolones and has a slightly higher MIC_50_ to metronidazole (Figure 2).

**Table 6:** Antimicrobial drug MICs for NAP 1/toxinotype III strains and other strains from case-patients with CDI, Monroe County, New York, USA, March 1–August 31, 2008*

Drug	NAP1/toxinotype III, μg/mL				Other strains, μg/mL		
	MIC_50_	MIC_90_	Range		MIC_50_	MIC_90_	Range
Clindamycin	<2	>32	<2 to>32		<2	>32	<2 to >32
Levofloxacin	>32	>32	<2 to >32		4	>32	<2 to >32
Moxifloxacin	16	>32	<2 to >32		<2	32	<2 to >32
Metronizadole	2	4	<0.5 to 4		<0.5	2	<0.5 to 2
Vancomycin	1	2	<0.5 to 2		1	2	<0.5 to 2

*NAP, North American pulsed-field; CDI, *Clostridium difficile* infection. Clinical and Laboratory Standards Institute interpretive criteria (sensitive/ intermediate/resistant): clindamycin: <2/4/>8 μg/mL; levofloxacin: not available; moxifloxacin: <2/4/>8 μg/mL; metronidazole: <8/16/>32 μg/mL; vancomycin: not available.

**Figure 2:** Metronidazole MICs (μg/mL) for North American pulsed-field 1 (NAP1) strains of *Clostridium difficile* compared with MICs for other strains, Monroe County, New York, USA, March 1–August 2008. tox, toxinotype.

## Discussion

CA-CDI was first described in the 1980s in patients receiving outpatient antimicrobial drug treatments (*27**–**29*). In 2005, the emergence of the NAP1 epidemic strain was associated with an increase in the incidence of HA-CDI and an increase in reports of CDI in low-risk populations, such as persons living in the community, children, and peripartum women (*4*). Our laboratory surveillance showed that 18% of the CDI cases were CA, a finding that is similar to other surveillance studies reporting percentages of 20%–30% (*9**,**30**–**32*). CA-CDI case-patients were younger and healthier than those with HA exposure (median age 53 vs. 72 years). Although 20% of CA case-patients had illness severe enough to require hospitalization, no CDI-related complications or deaths were reported.

In a population-based surveillance study in Durham North Carolina, USA, in 2005 (*9*), 59% of the CA-CDI case-patients required hospitalization, and 15% reported an emergency department visit. Similar to our findings, none of those case-patients required admission to intensive care units or surgical interventions, such as colectomy. Surveillance findings for CA-CDI in Connecticut, USA, in 2006 (*13*) showed that 111 (46%) of 241 CA-CDI case-patients required hospitalization, 29 (12%) required admission to intensive care units, 5 (2%) had toxic megacolon or colectomy, and 5 (2%) died of CDI complications. However, surveillance in Connecticut was conducted on the basis of preferential reporting by physicians and infection prevention specialists, which may have resulted in identification of the most severe disease and hospitalized case-patients. In addition, interviews were not performed to confirm lack of health care exposure.

We observed that 76% of CA-CDI case-patients were exposed to antimicrobial drugs in the 12 weeks before diagnosis. This percentage is higher than previously reported estimates of 40%–61% (*9**,**10**,**13**,**14**,**31**,**33**,**34*) and may reflect more complete information obtained during detailed case interviews. For example, several patients received antimicrobial drugs from their dentist, and such information is likely unavailable in outpatient medical records. These drugs were prescribed for common outpatient indications, and several patients received clindamycin for dental prophylaxis or infection. The role of PPI in CA-CDI remains controversial. Some studies have reported increased risk for disease associated with their use (*12**,**35*). Twenty-six percent of CA-CDI case-patients interviewed reported PPI use. However, it is difficult to assess if this is a major risk without a control group comparison.

Molecular testing showed a similar distribution of strains between HA and CA cases, and the percentage of cases with the NAP1 epidemic strain ranged from 46% in HA cases to 32% in CA-CDI cases. The percentage of CA cases with the NAP1 strain was similar to that in other reports (18%–37%) (*8**,**36**–**38*). Similar strain distribution in health care facilities and the community suggests that in contrast to the emergence of CA-methicillin-resistant *S*. *aureus* strains, there was no preferential transmission of particular strains within or outside the health care setting. Health care facilities might act as a reservoir for CA disease or that the community might act as a reservoir for HA-associated disease.

Our study examined potential exposure routes for *C. difficile* acquisition in the community. There are 4 postulated sources for exposure to *C. difficile* spores (*39*): consumption of contaminated food and water, animal-to-person contact, person-to-person contact, and environment-to-person contact. Foodborne acquisition has been hypothesized as a source of CA infections on the basis of recovery of *C. difficile* spores from food products and similarities between strains recovered from animals and those known to cause disease in humans (*15**–**19*). However, there is currently insufficient evidence to support foodborne acquisition as a common source of CA-CDI (*7*).

We assessed food and animal exposure during interviews with CA-CDI case-patients and did not find any specific association. However, we were unable to compare our observations with food and animal exposure patterns among persons in the community without CDI. Other possible sources of exposures include environments contaminated by *C. difficile* spores, such as hospitals and long-term care facilities; 21% of CA-CDI case-patients reported visiting or accompanying a family member to a health care facility in the 12 weeks before diagnosis. Contact with an ill or *C. difficile*–colonized family member or a household member who worked in a health care setting (i.e., someone who might have carried *C. difficile* spores on their hands or clothes) is another possible exposure. Two case-patients reported that a family member had diarrhea or was given a diagnosis of CDI, and several had a household member who worked in a health care setting. We also observed an excellent *C. difficile* recovery rate from refrigerated stool swabs, indicating that this method could be used in epidemiologic studies in which storage and processing of *C. difficile* specimens are required.

Our findings need to be interpreted in light of several limitations. We were unable to calculate the incidence of CA-CDI because surveillance did not include all laboratories servicing the Monroe County population. This study is descriptive, and the lack of a control group prevents us from estimating the risk for various exposures in development of CDI. At the time of this study, diagnosis of *C. difficile* relied on testing with the toxins A and B enzyme immunoassay, which has a sensitivity of 60%–90% and specificity of 90%–95%. In low- prevalence populations, such as outpatients, the positive predictive value is low and the likelihood of false-positive results is higher, which might have biased some results by including patients who did not have CDI (*40*). However, this bias was minimized by laboratory refusal of formed (i.e., nondiarrheal) fecal specimens and exclusion of cases without diarrheal symptoms. We did not review medical records from physician and dental offices. Therefore, patient-reported antimicrobial drug and PPI use was not confirmed. We attempted to interview all persons with potential CA-CDI but were unable to do so in 29% of the cases. These cases were defined as probable CA and included in our clinical summary. The small number of CA-CDI cases and isolates also limited our capacity to assess difference between NAP1 and other strains in severity and outcome of CDI.

In conclusion, CA-CDI represented 18% of CDI cases in Monroe County. CA-CDI case-patients were younger and healthier than HA-CDI case-patients. Use of antimicrobial drugs in outpatient settings remains a serious exposure, and even limited exposure to the health care environment or to persons in contact with health care facilities might play a crucial role in acquisition of CDI in the community. Prevention of CA-CDI will require further studies to understand risk factors leading to CDI in patients not exposed to antimicrobial drugs and the role of various potential exposures to *C. difficile*, such as food, animals, and household environment. Our results suggest that educating outpatient clinicians, including dentists, about the risk for community-associated CDI following use of oral antimicrobial drugs and that promoting judicious use of these drugs are potentially important interventions for the prevention of CDI in the outpatient setting.

## References

[1] Freeman J, Bauer MP, Baines SD, Corver J, Fawley WN, Goorhuis B, The changing epidemiology of *Clostridium difficile* infections. Clin Microbiol Rev. 2010;23:529–49. 10.1128/CMR.00082-0920610822PMC2901659

[2] Zilberberg MD, Shorr AF, Kollef MH. Increase in adult *Clostridium difficile*–related hospitalizations and case-fatality rate, United States, 2000–2005. Emerg Infect Dis. 2008;14:929–31. 10.3201/eid1406.07144718507904PMC2600276

[3] Kuijper EJ, Wilcox MH. Decreased effectiveness of metronidazole for the treatment of *Clostridium difficile* infection? Clin Infect Dis. 2008;47:63–5. 10.1086/58829418491959

[4] Centers for Disease Control and Prevention. Severe *Clostridium difficile*–associated disease in populations previously at low risk—four states, 2005. MMWR Morb Mortal Wkly Rep. 2005;54:1201–5.16319813

[5] McDonald LC, Killgore GE, Thompson A, Owens RC Jr, Kazakova SV, Sambol SP, An epidemic, toxin gene–variant strain of *Clostridium difficile.* N Engl J Med. 2005;353:2433–41. 10.1056/NEJMoa05159016322603

[6] Pépin J, Saheb N, Coulombe MA, Alary ME, Corriveau MP, Authier S, Emergence of fluoroquinolones as the predominant risk factor for *Clostridium difficile*–associated diarrhea: a cohort study during an epidemic in Quebec. Clin Infect Dis. 2005;41:1254–60. 10.1086/49698616206099

[7] Gould LH, Limbago B. *Clostridium difficile* in food and domestic animals: a new foodborne pathogen? Clin Infect Dis. 2010;51:577–82. 10.1086/65569220642351

[8] Limbago BM, Long CM, Thompson AD, Killgore GE, Hannett GE, Havill NL, *Clostridium difficile* strains from community-associated infections. J Clin Microbiol. 2009;47:3004–7. 10.1128/JCM.00964-0919571021PMC2738099

[9] Kutty PK, Woods CW, Sena AC, Benoit SR, Naggie S, Frederick J, Risk factors for and estimated incidence of community-associated *Clostridium difficile* infection, North Carolina, USA. Emerg Infect Dis. 2010;16:197–204.2011354710.3201/eid1602.090953PMC2958012

[10] Bauer MP, Veenendaal D, Verhoef L, Bloembergen P, van Dissel JT, Kuijper EJ. Clinical and microbiological characteristics of community-onset *Clostridium difficile* infection in the Netherlands. Clin Microbiol Infect. 2009;15:1087–92. 10.1111/j.1469-0691.2009.02853.x19624512

[11] Dial S, Delaney JA, Barkun AN, Suissa S. Use of gastric acid-suppressive agents and the risk of community-acquired *Clostridium difficile*–associated disease. JAMA. 2005;294:2989–95. 10.1001/jama.294.23.298916414946

[12] Delaney JA, Dial S, Barkun A, Suissa S. Antimicrobial drugs and community-acquired *Clostridium difficile*–associated disease, UK. Emerg Infect Dis. 2007;13:761–3.1755326010.3201/eid1305.061124PMC2738472

[13] Centers for Disease Control and Prevention. Surveillance for community-associated *Clostridium difficile—*Connecticut, 2006. MMWR Morb Mortal Wkly Rep. 2008;57:340–3.18385641

[14] Wilcox MH, Mooney L, Bendall R, Settle CD, Fawley WN. A case-control study of community-associated *Clostridium difficile* infection. J Antimicrob Chemother. 2008;62:388–96. 10.1093/jac/dkn16318434341

[15] Rodriguez-Palacios A, Staempfli HR, Duffield T, Weese JS. *Clostridium difficile* in retail ground meat, Canada. Emerg Infect Dis. 2007;13:485–7. 10.3201/eid1303.06098817552108PMC2725909

[16] Songer JG, Trinh HT, Killgore GE, Thompson AD, McDonald LC, Limbago BM. *Clostridium difficile* in retail meat products, USA, 2007. Emerg Infect Dis. 2009;15:819–21. 10.3201/eid1505.08107119402980PMC2687047

[17] Metcalf DS, Costa MC, Dew WM, Weese JS. *Clostridium difficile* in vegetables, Canada. Lett Appl Microbiol. 2010;51:600–2. 10.1111/j.1472-765X.2010.02933.x21069911

[18] Metcalf D, Reid-Smith RJ, Avery BP, Weese JS. Prevalence of *Clostridium difficile* in retail pork. Can Vet J. 2010;51:873–6.21037888PMC2905007

[19] Weese JS, Reid-Smith RJ, Avery BP, Rousseau J. Detection and characterization of *Clostridium difficile* in retail chicken. Lett Appl Microbiol. 2010;50:362–5. 10.1111/j.1472-765X.2010.02802.x20102510

[20] McDonald LC, Coignard B, Dubberke E, Song X, Horan T, Kutty PK, Recommendations for surveillance of *Clostridium difficile*–associated disease. Infect Control Hosp Epidemiol. 2007;28:140–5. 10.1086/51179817265394

[21] George WL, Sutter VL, Citron D, Finegold SM. Selective and differential medium for isolation of *Clostridium difficile.* J Clin Microbiol. 1979;9:214–9.42954210.1128/jcm.9.2.214-219.1979PMC272994

[22] Arroyo LG, Rousseau J, Willey BM, Low DE, Staempfli H, McGeer A, Use of a selective enrichment broth to recover *Clostridium difficile* from stool swabs stored under different conditions. J Clin Microbiol. 2005;43:5341–3. 10.1128/JCM.43.10.5341-5343.200516208013PMC1248507

[23] Rupnik M, Avesani V, Janc M, von Eichel-Streiber C, Delmee M. A novel toxinotyping scheme and correlation of toxinotypes with serogroups of *Clostridium difficile* isolates. J Clin Microbiol. 1998;36:2240–7.966599910.1128/jcm.36.8.2240-2247.1998PMC105025

[24] Killgore G, Thompson A, Johnson S, Brazier J, Kuijper E, Pepin J, Comparison of seven techniques for typing international epidemic strains of *Clostridium difficile*: restriction endonuclease analysis, pulsed-field gel electrophoresis, PCR-ribotyping, multilocus sequence typing, multilocus variable-number tandem-repeat analysis, amplified fragment length polymorphism, and surface layer protein A gene sequence typing. J Clin Microbiol. 2008;46:431–7. 10.1128/JCM.01484-0718039796PMC2238077

[25] Clinical and Laboratory Standards Institute (CLSI). Methods for antimicrobial susceptibility testing of anaerobic bacteria. Approved standard. 7th edition. CLSI document M11–A7. Wayne (PA): The Institute; 2007.

[26] Jhung MA, Thompson AD, Killgore GE, Zukowski WE, Songer G, Warny M, Toxinotype V *Clostridium difficile* in humans and food animals. Emerg Infect Dis. 2008;14:1039–45. 10.3201/eid1407.07164118598622PMC2630049

[27] Stergachis A, Perera DR, Schnell MM, Jick H. Antibiotic-associated colitis. West J Med. 1984;140:217–9.6730468PMC1021599

[28] Hirschhorn LR, Trnka Y, Onderdonk A, Lee ML, Platt R. Epidemiology of community-acquired *Clostridium difficile*–associated diarrhea. J Infect Dis. 1994;169:127–33. 10.1093/infdis/169.1.1278277174

[29] Levy DG, Stergachis A, McFarland LV, Van Vorst K, Graham DJ, Johnson ES, Antibiotics and *Clostridium difficile* diarrhea in the ambulatory care setting. Clin Ther. 2000;22:91–102. 10.1016/S0149-2918(00)87980-110688393

[30] Norén T, Akerlund T, Back E, Sjoberg L, Persson I, Alriksson I, Molecular epidemiology of hospital-associated and community-acquired *Clostridium difficile* infection in a Swedish county. J Clin Microbiol. 2004;42:3635–43. 10.1128/JCM.42.8.3635-3643.200415297509PMC497655

[31] Naggie S, Frederick J, Pien BC, Miller BA, Provenzale DT, Goldberg KC, Community-associated *Clostridium difficile* infection: experience of a veteran affairs medical center in southeastern USA. Infection. 2010;38:297–300. 10.1007/s15010-010-0025-020454827

[32] Karlström O, Fryklund B, Tullus K, Burman LG. A prospective nationwide study of *Clostridium difficile*–associated diarrhea in Sweden. The Swedish *C. difficile* study group. Clin Infect Dis. 1998;26:141–5.945552310.1086/516277

[33] Dial S, Kezouh A, Dascal A, Barkun A, Suissa S. Patterns of antibiotic use and risk of hospital admission because of *Clostridium difficile* infection. CMAJ. 2008;179:767–72. 10.1503/cmaj.07181218838451PMC2553880

[34] Naggie S, Miller BA, Zuzak KB, Pence BW, Mayo AJ, Nicholson BP, A case–control study of community-associated *Clostridium difficile* infection: no role for proton pump inhibitors. Am J Med. 2011;124:276.e1–7. 10.1016/j.amjmed.2010.10.01321396512

[35] Dial S, Delaney JA, Barkun AN, Suissa S. Use of gastric acid-suppressive agents and the risk of community-acquired *Clostridium difficile*–associated disease. JAMA. 2005;294:2989–95. 10.1001/jama.294.23.298916414946

[36] Bignardi GE, Settle C. Different ribotypes in community-acquired *Clostridium difficile.* J Hosp Infect. 2008;70:96–8. 10.1016/j.jhin.2008.04.00318511151

[37] MacCannell DR, Louie TJ, Gregson DB, Laverdiere M, Labbe AC, Laing F, Molecular analysis of *Clostridium difficile* PCR ribotype 027 isolates from eastern and western Canada. J Clin Microbiol. 2006;44:2147–52. 10.1128/JCM.02563-0516757612PMC1489423

[38] Warny M, Pepin J, Fang A, Killgore G, Thompson A, Brazier J, Toxin production by an emerging strain of *Clostridium difficile* associated with outbreaks of severe disease in North America and Europe. Lancet. 2005;366:1079–84. 10.1016/S0140-6736(05)67420-X16182895

[39] Otten AM, Reid-Smith RJ, Fazil A, Weese JS. Disease transmission model for community-associated *Clostridium difficile* infection. Epidemiol Infect. 2010;138:907–14. 10.1017/S095026880999164620092667

[40] Planche T, Aghaizu A, Holliman R, Riley P, Poloniecki J, Breathnach A, Diagnosis of *Clostridium difficile* infection by toxin detection kits: a systematic review. Lancet Infect Dis. 2008;8:777–84. 10.1016/S1473-3099(08)70233-018977696

# Research article: Community-associated *Clostridium difficile* Infections, Monroe County, New York, USA

**DOI:** 10.3201/eid1803.102023

**Authors:** Ghinwa Dumyati, Vanessa Stevens, George E. Hannett, Angela D. Thompson, Cherie Long, Duncan MacCannell, Brandi Limbago

**Affiliations:** University of Rochester, Rochester, New York, USA (G. Dumyati, V. Stevens);; State University of New York at Buffalo, Buffalo, New York, USA (V. Stevens);; New York State Department of Health, Albany, New York, USA (G.E. Hannett);; Centers for Disease Control and Prevention, Atlanta, Georgia, USA (A.D. Thompson, C. Long, D. MacCannell, B. Limbago)

## Earning CME Credit

To obtain credit, you should first read the journal article. After reading the article, you should be able to answer the following, related, multiple-choice questions. To complete the questions (with a minimum 70% passing score) and earn continuing medical education (CME) credit, please go to www.medscape.org/journal/eid. Credit cannot be obtained for tests completed on paper, although you may use the worksheet below to keep a record of your answers. You must be a registered user on Medscape.org. If you are not registered on Medscape.org, please click on the New Users: Free Registration link on the left hand side of the website to register. Only one answer is correct for each question. Once you successfully answer all post-test questions you will be able to view and/or print your certificate. For questions regarding the content of this activity, contact the accredited provider, CME@medscape.net. For technical assistance, contact CME@webmd.net. American Medical Association’s Physician’s Recognition Award (AMA PRA) credits are accepted in the US as evidence of participation in CME activities. For further information on this award, please refer to http://www.ama-assn.org/ama/pub/category/2922.html. The AMA has determined that physicians not licensed in the US who participate in this CME activity are eligible for *AMA PRA Category 1 Credits*™. Through agreements that the AMA has made with agencies in some countries, AMA PRA credit may be acceptable as evidence of participation in CME activities. If you are not licensed in the US, please complete the questions online, print the certificate and present it to your national medical association for review.

## Article Title: Community-associated *Clostridium difficile* Infections, Monroe County, New York, USA

## CME Questions

**1. On the basis of the study by Dumyati and colleagues, which of the following statements about the relative burden of and potential risk factors for community-associated (CA) *Clostridium difficile* infection (CDI) disease is most likely correct?**A. CA-CDI accounted for about half of CDI cases in Monroe County, New York, in 2008B. The proportion of CA-CDI in this study was much higher than that reported in other recent surveillance studiesC. About three quarters of patients with CA-CDI had taken antibiotics in the 12 weeks before diagnosisD. About half of patients with CA-CDI reported proton pump inhibitor use**2. On the basis of the study by Dumyati and colleagues, which of the following statements about clinical characteristics of patients with CA disease is most likely correct?**A. About one fifth of the hospitalized CA patients died or had serious complicationsB. In addition to diarrhea, 86% reported abdominal pain, 48% nausea, 40% fever, and 17% bloody stoolC. CA-CDI patients were older than HA-CDI patientsD. Molecular testing showed significantly different distribution of strains between HA-CDI and CA-CDI**3. You are a public health official asked to consult on strategies for CA-CDI prevention and surveillance. On the basis of the review by Dumyati and colleagues, which of the following statements would most likely appear in your report?**A. Reducing exposure to specific foods and animals is a viable strategyB. Outpatient clinicians should be educated about the risk for CA-CDI following oral antibiotic use and about the need for judicious antibiotic useC. Risk factors leading to CDI in patients not exposed to antibiotics are well definedD. *C. difficile* recovery rate from refrigerated stool swabs is poor

## Activity Evaluation

**1:** The activity supported the learning objectives.

Strongly Disagree				Strongly Agree
1	2	3	4	5
**2. The material was organized clearly for learning to occur.**				
Strongly Disagree				Strongly Agree
1	2	3	4	5
**3. The content learned from this activity will impact my practice.**				
Strongly Disagree				Strongly Agree
1	2	3	4	5
**4. The activity was presented objectively and free of commercial bias.**				
Strongly Disagree				Strongly Agree
1	2	3	4	5
